# Microfluidic Fabrication of Morphology-Controlled Polymeric Microspheres of Blends of Poly(4-butyltriphenylamine) and Poly(methyl methacrylate)

**DOI:** 10.3390/ma11040582

**Published:** 2018-04-10

**Authors:** Saki Yoshida, Shu Kikuchi, Shinji Kanehashi, Kazuo Okamoto, Kenji Ogino

**Affiliations:** 1 Graduate School of Bio-Applications Systems Engineering, Tokyo University of Agriculture and Technology, Koganei, Tokyo 184-8588, Japan; s169262q@st.go.tuat.ac.jp (S.Y.); s166544q@st.go.tuat.ac.jp (S.K.); kanehasi@cc.tuat.ac.jp (S.K.); 2 Ushio Chemix Co., Ltd., Kakegawa, Shizuoka 437-1302, Japan; k.okamoto.1618@gmail.com

**Keywords:** microsphere, morphology, polymer blend, microfluidic, microreactor, poly(4-butyltriphenylamine)

## Abstract

Multicomponent polymer particles with specific morphology are promising materials exhibiting novel functionality which cannot be obtained with single-component polymer particles. Particularly, the preparation of such kinds of polymer particles involving electrically or optically active conjugated polymers with uniform size is a challenging subject due to their intense demands. Here, microspheres of binary polymer blend consisting of poly(4-butyltriphenylamine) (PBTPA)/poly(methyl methacrylate) (PMMA) (1:1 in weight) were produced via a microfluidic emulsification with a Y-shaped microreactor, and a subsequent solvent evaporation method. The flow rate of the dispersed phase (polymer solution) was fixed to 7 µL/min, and 140 or 700 µL/min of the flow rate of the continuous phase (aqueous 0.6 wt % of poly(vinyl alcohol) (PVA) solution) was utilized to produce the dispersion with different diameter. The concentration of dispersed phase was adjusted to 0.1 or 1.0 *w/v*%. Core-shell, Janus and dumbbell type microspheres were obtained dependent on the flow rate of continuous phase. Incomplete core-shell type microspheres were produced for the blend involving low molecular weight PMMA. Complex Janus and core-shell type microspheres were fabricated by the addition of sodium dodecyl sulfate (SDS) to continuous phase. It is found that final morphologies are strongly dependent on the initial conditions of dispersion including the particle size suggesting that the morphologies are governed by the kinetical factors together with the conventionally accepted thermodynamic ones.

## 1. Introduction

Polymer microspheres with various functionalities have been utilized in the fields of medicine and electric devices [1,2]. The function of polymer microspheres originates both from chemical and physical properties of polymeric components. As physical properties, the porosity, the surface area, and mechanical strength play important roles in the field of the solid catalysts and the various types of supports. In addition, the shape, the size, and the internal morphology are also important factors to create and improve the novel functions [3,4,5,6]. Among the types of particles, composite polymer particles consisting of two or more kinds of polymer components have been known to exhibit special functions. Since most of the different polymers are incompatible with each other except for rare combinations, the multiphases comprising the respective polymer appear during the solvent evaporation form the homogeneous solution. From this heterogeneous nature, it is known that functionality which cannot be obtained with homogeneous polymer particles can emerge. Much research has been conducted utilizing phase separation to fabricate microspheres with the target structure via the solvent evaporation method [7,8,9,10,11]. For example, Janus-type microspheres with electrical anisotropy can be utilized in electronic paper [2]. The control of layer thickness of onion-like multilayered microspheres [12] potentially makes it possible to manipulate light in optical applications. 

Our research group previously reported the fabrication of microspheres consisting of blend of poly(4-butyltriphenylamine) (PBTPA) and poly(methyl methacrylate) (PMMA) via the solvent evaporation method and investigated the effect of molecular weight of PBTPA on the morphology [13]. PBTPA has a semi-conjugated structure in which 4-butyltriphenylamine units are directly linked together and exhibits excellent hole-transporting properties. It was also found that PBTPA is a promising optical material due to the superior transparency in the visible light and high refractive index of 1.71 at 633 nm [14]. According to the previous paper, the structure of microspheres was strongly dependent on the molecular weight of PBTPA. For example, in the case of low molecular weight PBTPA (*M*_n_ = 4000), Janus-type morphology was observed for the blend composed of equal weight ratio with PMMA from the chloroform solution. Morphology changed to dumbbell type when PBTPA with high molecular weight (*M*_n_ = 10,000) was used. In this previous study, however the initial dispersion of chloroform solution was prepared with a homogenizer, and the size of the resulting dispersion was ununiform (2–10 μm). Uniform particles have been obtained by the combination of the solvent evaporation method with other techniques generating uniform solution droplets [15,16]. Among them, much attention has been paid to microfluidic technique from both the fundamental and practical viewpoints, since it can afford uniform particles with a diameter with several tens of micrometer [17,18]. However, most of microfluidic approaches have been limited to the conventional polymers, and few studies have been carried out concerned with the functional conjugated polymers such as PBTPA. 

In this paper, we report microfluidic approach to fabricate microspheres with a variety of morphologies based on PBTPA/PMMA binary blends, where the combination of the microfluidic emulsification with a Y-shaped microreactor, and the subsequent solvent evaporation method was utilized. Effects of the flow rate of continuous phase, the initial concentration of dispersed phase, the molecular weight of PMMA and the addition of the surfactant to the continuous phase on the morphologies were investigated. It is found that final morphologies are strongly dependent on the initial conditions of dispersion including the particle size suggesting that the morphologies are governed by the kinetical factors together with the conventionally accepted thermodynamic ones. Few approaches have been reported for the interpretation of morphology based on both the kinetical and thermodynamical points of view. 

## 2. Materials and Methods

### 2.1. Materials

PBTPA homopolymer (number average molecular weight (*M*_n_) = 9100, polydispersity index (PDI) = 1.80) was synthesized via palladium-catalyzed C-N coupling polymerization [19]. The number average molecular weight (*M*_n_) was determined by gel permeation chromatography (GPC) calibrated with polystyrene standards. Differential scanning calorimetry (DSC) analysis was performed under nitrogen atmosphere at heating and cooling rates of 10 °C/min on a DSC-8230 (Rigaku, Tokyo, Japan). As reported, obtained PBTPA showed amorphous nature with a glass transition temperature of 215 °C [19]. Two poly(methyl methacrylate)s (PMMAs) with different molecular weight (PMMA-L; *M*_n_ = 15,000, and PMMA-H; *M*_n_ = 120,000) was supplied by Wako Pure Chemical Industries (Osaka, Japan) as primary ingredients of the microspheres. DSC measurements revealed that both PMMAs showed a glass transition at 105 °C. Equal weight of two polymers were dissolved in chlorobenzene (Wako Pure Chemical Industries, Osaka, Japan) to have concentration of 0.1 or 1.0 *w*/*v*% for total polymers; the resulting solution was used as a dispersed phase of emulsion. Poly(vinyl alcohol) (PVA) (PVA224, Kuraray, Tokyo, Japan), and sodium dodecyl sulfate (SDS) (Wako Pure Chemical Industries, Osaka, Japan) was used as a water-soluble stabilizer, and a surfactant, respectively. 

### 2.2. Microfluidic Device and Particle Fabrication

**Figure 1:**
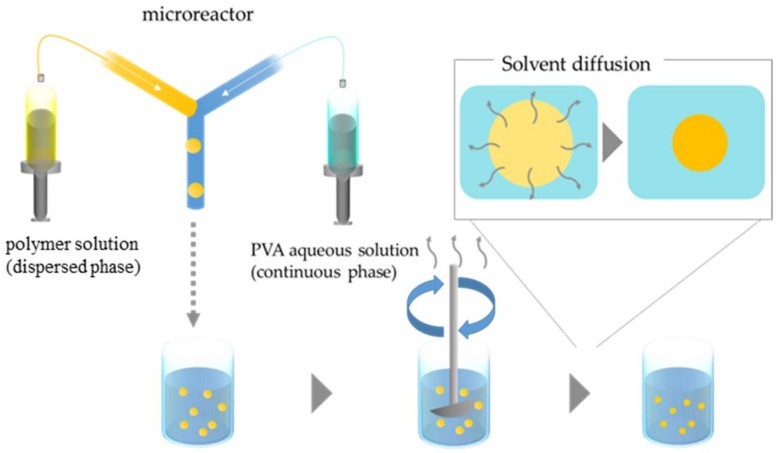
Schematic illustration of procedures to produce microspheres by combining microfluidic emulsification and solvent evaporation.

To produce microspheres of binary blend, oil-in-water (O/W) emulsion droplets were prepared using Y-shaped microreactor (YMC, Kyoto, Japan) (Figure 1). The Y-channel for generating emulsions in the stainless steel mixer (30 mm × 30 mm, 1.3 mm in thickness) is 0.5 mm wide and 0.1 mm deep. The dispersed and continuous phases were delivered from two gastight Hamilton syringes (Hamilton Company, Reno, NV, USA) with Lure-Lock fitting mounted syringe pumps onto the microreactor. The flow rate of the dispersed phase (polymer solution) was fixed to 7 µL/min, and 140 or 700 µL/min of the flow rate of the continuous phase (aqueous 0.6 wt % of PVA solution) was examined. The concentration of dispersed phase was adjusted to 0.1 or 1.0 *w*/*v%*. In some experiments, sodium dodecyl sulfate (0.06 wt %) was added to the aqueous phase. The weight ratio of PBTPA to PMMA was kept constant at 1:1. Table 1 represents the preparation conditions of emulsions.

The droplets were collected in a three-necked 300 mL flask equipped with a mechanical stirrer without plugs containing 200 mL of aqueous solution, which was the same as the continuous phase over 2 h at room temperature. The resulting dispersion was gently agitated with a mechanical stirrer at 70 rpm for 120 h to evaporate chlorobenzene. The produced microspheres were washed with distilled water four times with a centrifugation process. The observations with scanning electron microscope (SEM, JSM-6510, JEOL, Tokyo, Japan) and transmission electron microscope (TEM, JEM2100, JEOL, Tokyo, Japan) were carried out to evaluate the morphologies. In some cases, microspheres were washed with acetone to remove PMMA to clarify the component in the phase-separated domain from SEM images since only PMMA is soluble in acetone. Specimens for SEM were prepared by putting one drop of methanol dispersing the particles on the sample stage, and then dried in air. The samples were coated by osmium tetraoxide (OsO_4_) using ion coater (Neoc-STB, Meiwafosis Co. Ltd., Tokyo, Japan) to avoid the charging. To prepare the specimens for TEM observation, microspheres were embedded in epoxy resin (a mixture of Quetol-812, dodecenyl succinic anhydride, methyl nadic anhydride and DMP-30), and processed into thin slice with 70 nm of thickness. To stain PBTPA domain, the specimens were exposed to the vapor of an aqueous RuO_4_ solution (0.5%) for 10 min in a sealed bottle at room temperature. From SEM images, the value of coefficient of variation (CV) was estimated by measuring the diameters of 100 particles.

## 3. Results

### 3.1. Effect of Flow Rate of Continuous Phase and Concentration of Dispersed Phase

**Figure 2:**
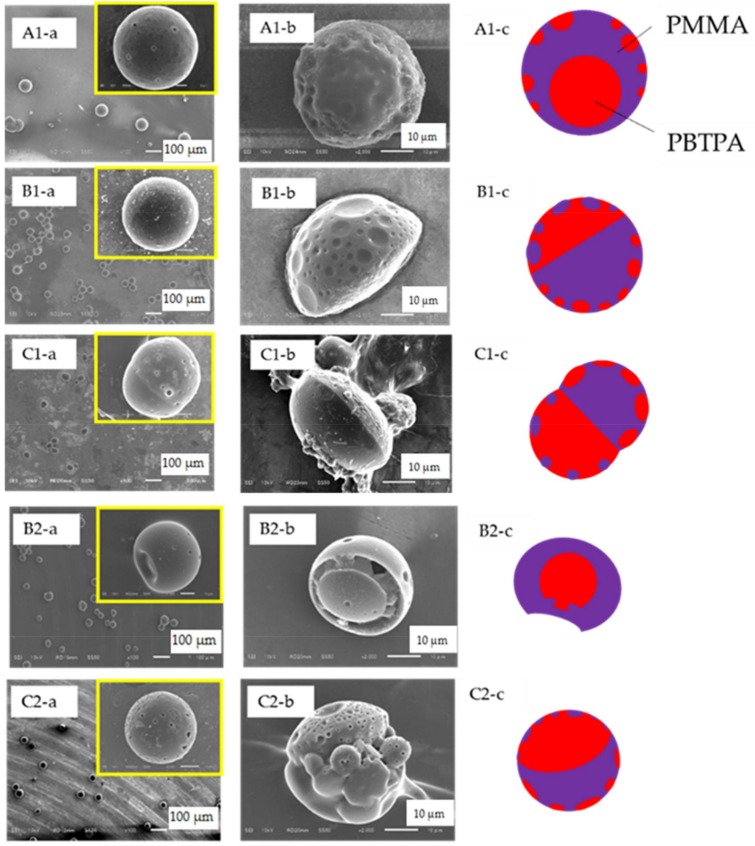
SEM images of microspheres of PBTPA/PMMA prepared with the conditions listed in Table 1 (A1, B1, B2, C1 and C2), (**a**) as-prepared, (**b**) after soaking with acetone, (**c**) schematic models.

The microspheres were successfully fabricated via the formation of dispersion with Y-shaped microfluidic system under several conditions, and the subsequent solvent evaporation. Figure 2 shows SEM images of obtained microspheres (a), those of microspheres washed with acetone (b) and schematic models (c) of polymer blend particles. 

In the fabrication condition represented as A1 in Table 1, core-shell type of microspheres (PMMA; shell, and PBTPA; core, each component was assigned from SEM images) were obtained. Average diameter was determined to 72.4 µm and CV was estimated to 13.1% from SEM image. Surface image suggests that smaller spherical PBTPA domains exist near the surface of the PMMA shell, which is confirmed by SEM and TEM observations (see Figure S1 in Supplementary Materials). When the flow rate of continuous phase increased to 700 µL/min, the morphology was changed to Janus type (B1 in Table 1), and the average diameter decreased to 44.9 µm (CV; 4.5%). As shown in B1 series of Figure 2, hemispheres with dents were obtained by washing with acetone, and the existence of dents suggests that small PMMA domains also locate on the surface of PBTPA hemisphere. Based on the assumption that the size of initially produced droplet decreases with the increase of flow rate of the continuous phase, it is suggested that the final morphology is dependent on the size of droplet. Furthermore, the increase of the flow rate of the continuous phase make the resulting microspheres more uniform. The uniformity suggests that the relatively uniform droplets are formed on the conditions we examined, and the droplet coalescence and breakage are negligible during the evaporation process. When the concentration of the dispersed phase decreased to 0.1 *w/v*%, dumbbell-like microspheres were produced (C1 series of Figure 2), where the average particle diameter is 51.9 µm, and CV is 8.5%. The decrease of concentration makes the droplet size smaller at the onset of liquid-liquid phase separation compared with conditions represented as A1. It is found that both the initial size and concentration affect the final morphology of microspheres.

### 3.2. Effect of Molecular Weight of PMMA

The effect of molecular weight of PMMA on the morphology was investigated using low molecular weight PMMA (PMMA-L) (conditions B2, and C2 in Table 1). By decreasing molecular weight of PMMA on the same conditions as A1, incomplete core-shell microspheres (PMMA; shell, and PBTPA; core, each component was assigned from SEM image) having a single dent were obtained on the condition B2. The unique morphology was observed for PBTPA rich domain. In the case of the condition C2, the dumbbell-like morphology changed to incomplete core-shell. It is known that interfacial tension between polymers increases with increasing molecular weight [19], resulting in the change of morphology. The decrease of molecular weight of PMMA also makes the polymer translational motion in droplet faster during phase separation compared with conditions B1, and C1. It is suggested that the final morphology is dependent on interfacial characteristics and/or mobility of polymer chain.

### 3.3. Effect of SDS

**Figure 3:**
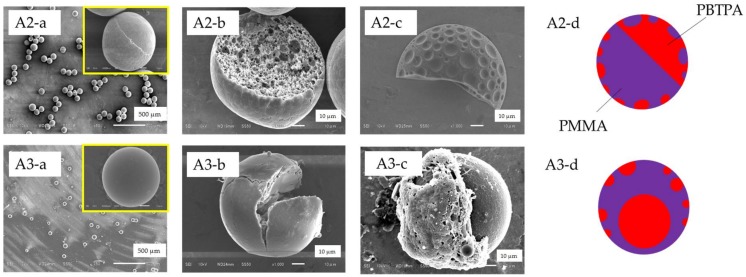
SEM images of microspheres of PBTPA/PMMA prepared with the conditions listed in Table 1 (A2 and A3), (**a**) as-prepared, (**b**) cracked, (**c**) after soaking with acetone, (**d**) schematic models.

To investigate the effect of a surfactant, SDS was added to the continuous phase. Figure 3 shows SEM images of as-prepared (a) and cracked (b) microspheres, and those of microspheres washed with acetone (c), and schematic models (d) in the addition of SDS. The cracking of microspheres was done by pressing the sample mounted on a glass slide with another glass slide. Fabrication condition (A2) where SDS (0.06 wt %) was added to the continuous phase, and other factors were the same as the condition A1 afforded microspheres with complex Janus structure where one polymer domains were dispersed in another polymer phase in each hemisphere. It is noteworthy that the PMMA rich domain is porous. Average diameter was determined to be 96.7 µm and CV was estimated to 3.1% from SEM image. Larger diameter compared with microspheres fabricated without SDS (A1) was probably ascribed to the porous nature of PMMA domain. When the rate of solvent evaporation was reduced by plugging the two mouths on the side necks of the three-necked 300 mL flask, the morphology was changed to core-shell microspheres (PMMA; non-porous shell, and PBTPA; core, each component was assigned from TEM image), and the average diameter decreased to 52.0 µm (CV; 13.7%) (A3 series in Figure 3). Suppression of the solvent evaporation also reduces the solvent diffusion from the droplets to the continuous phase allowing prolong time for the construction of final morphology leading to the thermodynamically stable structure. 

## 4. Discussion

### 4.1. Phase Separation of Polymers in Emulsion Drops

When two immiscible polymers, PMMA and PBTPA, are dissolved in chlorobenzene, they are fully mixed in the initial homogeneous oil droplets because of the entropic gain (ΔS_m_ > 0). Evaporation affords the binary blend microspheres with uniform size if the uniform droplets are formed by a microfluidic process, and the droplet coalescence and breakage are negligible during the evaporation process. As the solvent is evaporated, the concentration of each polymer increases and reaches the critical value at which the phase separation starts. The phase separation starts via a spinodal decomposition or a nucleation and growth mechanism. Independent of initial process of phase separation, the subsequent coarsening and coalescence of domains occur to decrease the interfacial energy leading to Janus morphology since the difference of interfacial energies of two different oil phases for the aqueous phase is small when enough of solvent remains.

**Figure 4:**
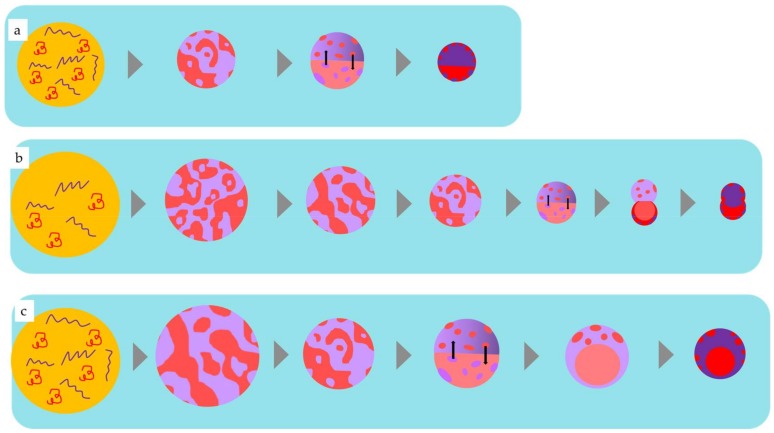
Plausible temporal evolution of phase separated morphology for PBTPA/PMMA composite microsphere (**a**) B1, (**b**) C1, (**c**) A1, red: PBTPA, blue: PMMA.

As the evaporation of solvent proceeds, the Janus morphology transforms to a core-shell one where the more hydrophilic domain (PMMA) is located at the interface with the aqueous phase to form the shell and the more hydrophobic one (PBTPA) to form the core. This transition accompanied with the translational movements of domains. Figure 4 schematically represents the mechanism for the formation of microspheres fabricated with different conditions. As the flow rate of the continuous phase increases, it is easily predicted to decrease the size of the initially formed droplet.

Taking the size of the final particle into consideration, the absolute amount of polymers in a single droplet increases in the order of a, b, and c in Figure 4. Assuming the onset of the phase separation is only dependent on the concentration of polymers, the critical size in which the phase separation starts is also in the same order as illustrated in Figure 4. Since the specific surface area is inversely proportional to the diameter, the increase of the diameter leads to the decrease of total diffusion rate of the solvent from the droplet to the aqueous. Delayed diffusion process results in the prolonged evaporation time. As mentioned above, at the initial stage of liquid-liquid phase separation, Janus morphology is favored. If the solvent evaporation process is sufficiently slow, the morphology changes to thermodynamically stable core-shell type as shown in Figure 4c. The smaller critical size as illustrated in Figure 4a led to Janus-type particles since the fast evaporation process did not afford enough time for the transformation. Consequently, kinetically controlled morphology is obtained. In the case of moderate critical size as shown in Figure 4b, the transformation is interrupted before it completes leading to the dumbbell-like morphology. 

**Figure 5:**
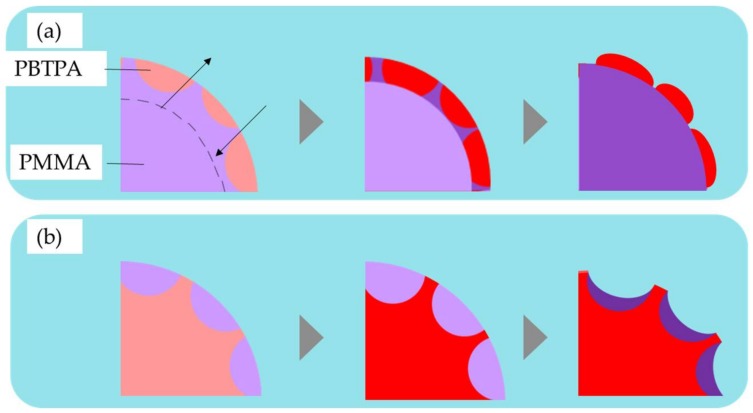
Formation of surface morphology of microspheres of PBTPA/PMMA blend (the surface consists mainly of (**a**) PMMA-rich phase, (**b**) PBTPA-rich phase).

On the surface of the resulting microspheres, there are circle domains of another components (see Figures S1 and S2 in Supplementary Materials). For example, in the PMMA-rich phase, the protrusion of PBTPA-rich phase was observed, while PMMA-rich domains in PBTPA-rich phase exists as dents. Figure 5 represents the mechanism for the formation of surface morphology. As discussed above, the coarsening and coalescence of domains after the initial phase separation occur to form Janus morphology. It is reasonable, however, that some domains especially near the surface are left behind since the domains near the surface solidify at an earlier stage than the those inside the droplets. Moreover, PBTPA is classified into semirigid polymers, and its cohesive power is stronger than that of PMMA. It is reasonable to consider that PBTPA solidifies earlier than PMMA. In this situation, more solvent distributes in PMMA-rich domain than PBTPA-rich domain. That is why the protrusion of PBTPA-rich domain, and the dent of PMMA-rich domain, are observed. 

### 4.2. Effect of Molecular Weight of PMMA

**Figure 6:**
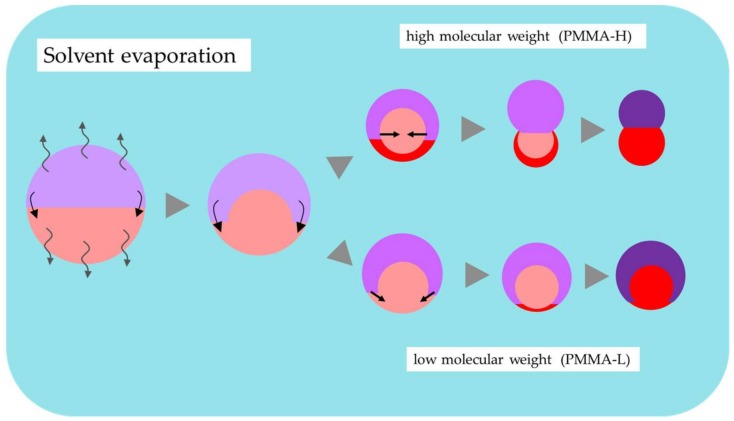
Illustration of the effect of molecular weight of PMMA in the formation of PBTPA/PMMA composite microsphere.

As shown in Figure 2, the morphology of the resulting microsphere is also dependent on the molecular weight of PMMA. Because of the complex morphology observed in condition B2, it is difficult to discuss how this morphology is established until now. Therefore, in this subsection, discussion is carried out for the difference of morphologies obtained in the conditions C1 and C2. As discussed in a previous subsection, the liquid-liquid phase separated droplets are stabilized as a Janus type since the interfacial tension between the solution of each polymer (PBTPA or PMMA) and the PVA aqueous solution is nearly equal. As the solvent diffuses and evaporates, the polymer content increases to form semi-solid phases. As shown in Figure 6, the concentrated PMMA-rich phase has higher hydrophilicity than the PBTPA-rich phase leading to the translational movements of the PMMA-rich phase to surround the PBTPA-rich phase. However, when the molecular weight of PMMA is high (PMMA-H), this motion is more restricted due to the high viscosity. Therefore, the PBTPA-rich phase keeps contact with PVA solution for longer time compared with the case of PMMA-L, leading to the faster evaporation or solidification of the PBTPA-rich phase. This solidification further restricts the translational motion of PMMA-rich phase resulting in the coverage of the PBTPA-rich phase by the PMMA-rich phase. In the case of PMMA-L, due to the faster translational motion of the PMMA-rich phase, it is considered that the PBTPA-rich phase is covered with the PMMA-rich phase before solidification of PBTPA-rich phase.

As described in Section 3.2, it is reported that interfacial tension between polymers increases with increasing molecular weight [20]. In the case of polystyrene/PMMA composite particles, the effect of the molecular weight is mainly discussed with the molecular weight dependence of the interfacial tension [8,9]. In this study, semirigid PBTPA is utilized as the counterpart. Because of its strong cohesive power, it can be assumed that the PBTPA-rich phase solidifies earlier than the PMMA-rich phase. Therefore, it is considered that the effect of molecular weight on the translational motion plays an important role in the determination of the morphology.

### 4.3. Effect of Surfactant

**Figure 7:**
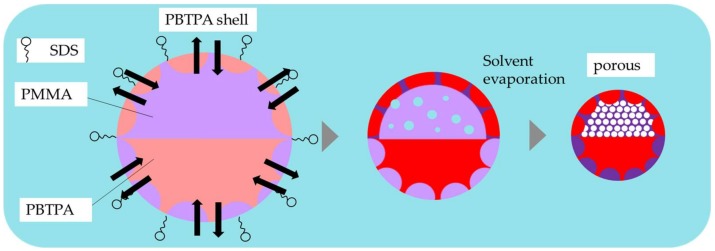
Illustration of the effect of SDS in the formation of PBTPA/PMMA composite microsphere.

A surfactant, SDS is an amphiphilic compound possessing both hydrophilic and hydrophobic moieties. While the difference of the interfacial energies of the solutions of PBTPA and PMMA is small governed by the nature of solvent (chlorobenzene), solidified PMMA exhibits higher interfacial energy due to its hydrophilic nature. SDS in the aqueous phase adsorbs on the surface of the oil droplet, leading to the reduction of the interfacial tension between oil and aqueous phases, and the difference of interfacial energies of both polymer solutions. Figure 7 illustrates the effect of SDS and the schematic representation of the formation of morphology. The addition of SDS afforded Janus-type morphology (A2) rather than core-shell morphology without SDS (A1). It is noteworthy that porous nature was observed in the PMMA rich hemisphere. Porous structure is ascribed to the formation of double emulsions. It is considered that the existence of SDS makes the intrusion of water into the oil phase during evaporation facile to form the water droplets inside the oil phase, and the solidification of polymers proceeded fast before the extrusion of water. It is reported that porous microspheres are obtained for amphiphilic poly(4-vinylpyridine)-b-poly{6-[4-(4-butyloxyphenylazo) phenoxy]hexyl methacrylate} by a solvent evaporation method, where the porous structure is also ascribed to the permeation of water to the oil-droplets [21]. Decrease of the evaporation rate by plugging the two mouths on the side necks of the three-necked flask made the microspheres non-porous and the morphology core-shell type. Prolonged diffusion time allowed water inside the droplet to extrude during the evaporation process resulting in the non-porous microspheres with core-shell morphology, which is thermodynamically more stable. 

## 5. Conclusions

We reported here the fabrication of uniform microspheres consisting of PBTPA and PMMA composite with various phase-separated morphologies utilizing the combination of microfluidic emulsification with Y-shaped microreactor and solvent evaporation. Coefficient of variation of all fabricated microspheres was below 14%. The flow rate of the continuous phase, and the initial concentration of polymers influence the final morphologies, which can be explained by the critical droplet size in which the phase separation starts. Lowering molecular weight of PMMA in dispersed phase changed the morphology from Janus to incomplete core-shell due to the faster translational motion of the PMMA-rich phase. These observations strongly suggest that the morphologies are governed by kinetical factors as well as conventionally accepted thermodynamic ones. The addition of SDS to continuous phase afforded the porous microspheres and changed the morphology to Janus type by the reduction of the interfacial tension between oil and aqueous phases, and the difference of interfacial energies of both polymer solutions. By slowing the evaporation, core-shell type, non-porous microspheres were obtained. Our microfluidic approach can be extended to fabricate the composite microspheres with more complicated or regularly phase-separated morphology. Therefore, it is convinced that the microfluidic technique will provide the desired morphologies suitable for electrical or optical applications. 

## Figures and Tables

**Table 1:** Microfluidic conditions for preparation of emulsions, and characteristics of resulting blend microspheres.

Condition	Continuous Phase ^1^		Dispersed Phase ^2^		Average Diameter (μm)	CV ^4^ (%)	Type
	Flow Rate (μL/min)	SDS (0.06 wt %)	Conc. of Polymers (wt %) ^3^	*M*_w_ of PMMA (/10^4^)			
A1	140	-	1.0	12	72.4	13.1	core-shell
A2 ^5^	140	+	1.0	12	96.7	3.1	Janus
A3 ^6^	140	+	1.0	12	52.0	13.7	core-shell
B1	700	-	1.0	12	44.9	4.5	Janus
B2	700	-	1.0	1.5	44.6	8.6	incomplete core-shell
C1	140	-	0.1	12	51.9	8.5	dumbbell
C2	140	-	0.1	1.5	39.7	11.3	incomplete core-shell

^1^ aqueous PVA solution (0.6 wt %), ^2^ flow rate of dispersed phase was fixed to 7 μL/min, ^3^ total concentration of PBTPA and PMMA, ^4^ coefficient of variation, ^5^ 7 days for solvent evaporation, ^6^ 13 days for solvent evaporation (the rate of solvent evaporation was reduced by plugging the two mouths on the side necks of the three-necked flask).

## Supplementary Materials

The following are available online at http://www.mdpi.com/1996-1944/11/4/582/s1, Figure S1: SEM (expanded) (a) and TEM (b) images of microspheres of PBTPA/PMMA prepared with the condition A1 as listed in Table 1, Figure S2: Surface SEM image of microspheres of PBTPA/PMMA prepared with the conditions B1(a) and C1(b) as listed in Table 1.
