# Supplementary material for: Microfluidic Fabrication of Morphology-Controlled Polymeric Microspheres of Blends of Poly(4-butyltriphenylamine) and Poly(methyl methacrylate)
Source: Materials (Basel). 2018 Apr 10;11(4):582. doi: 10.3390/ma11040582 (PMC5951466; doi:10.3390/ma11040582)
Supplement: Supplementary file 1 [file materials-11-00582-s001.pdf]

Supplementary Materials

Microfluidic Fabrication of Morphology-controlled Polymeric Microspheres of Blends of Poly(4-butyltriphenylamine) and Poly(methyl methacrylate)

Saki Yoshida[1], Shu Kikuchi[1], Shinji Kanehashi[1], Kazuo Okamoto[2], and Kenji Ogino[1]*

[1]Graduate School of Bio-Applications and Systems Engineering,
Tokyo University of Agriculture and Technology,
2-24-16 Nakacho, Koganei, Tokyo 184-8588, Japan

[2]Ushio Chemix Co., Ltd., Kakegawa, Shizuoka 437-1302, Japan

* Correspondence: kogino@cc.tuat.ac.jp; Tel/Fax: +81-42-388-7404

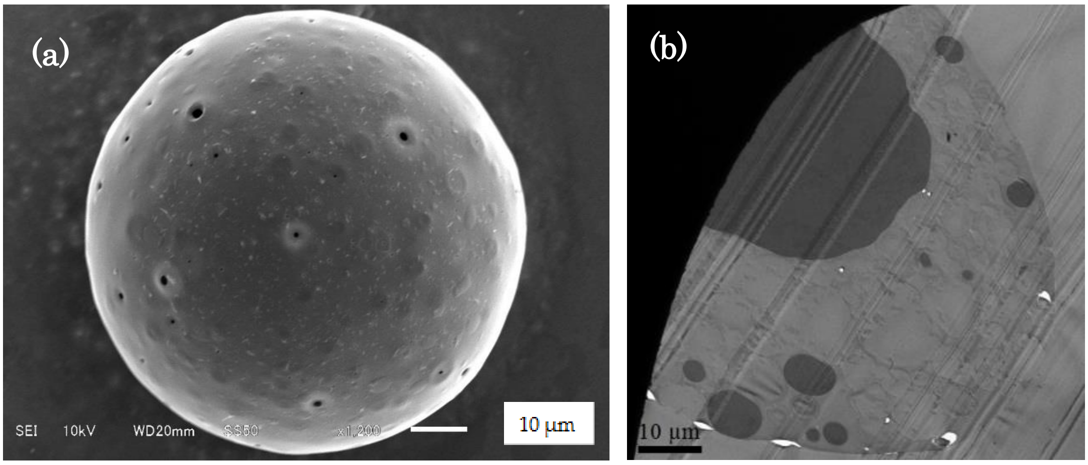

Figure S1. SEM (expanded) (a) and TEM (b) images of microspheres of PBTPA / PMMA prepared with the condition A1 as listed in Table 1.

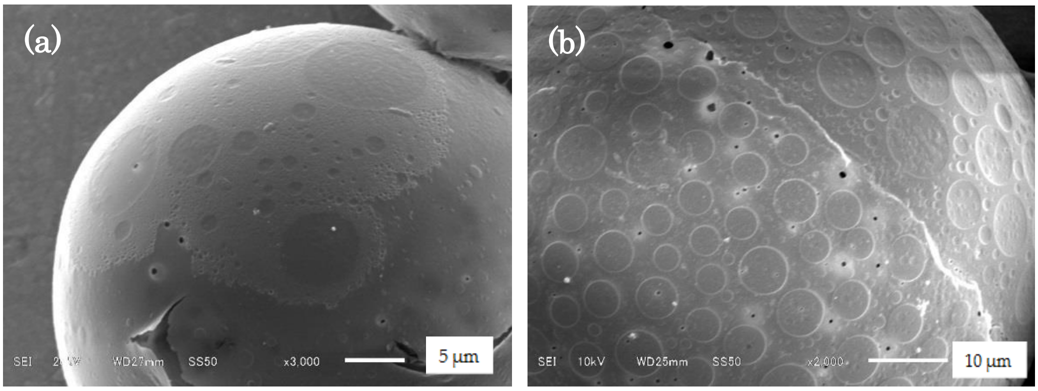

Figure S2. Surface SEM image of microspheres of PBTPA / PMMA prepared with the conditions B1(a) and C1(b) as listed in Table 1.
